# Factors Affecting Ankle Support Device Usage in Young Basketball Players

**DOI:** 10.3390/jcm2020022

**Published:** 2013-05-10

**Authors:** Michael D. Cusimano, Ahmed Faress, Wilson P. Luong, Khizer Amin, Joanne Eid, Tamer Abdelshaheed, Kelly Russell

**Affiliations:** Injury Prevention Research Office, Keenan Research Centre, Li Ka Shing Knowledge Institute, St Michael’s Hospital, Toronto M5B 1W8, Canada; E-Mails: a.faress@utoronto.ca (A.F.); wilson.p.luong@gmail.com (W.P.L.); khizeramin@gmail.com (K.A.); joanne.eid@hotmail.com (J.E.); tamer.abdelshaheed@utoronto.ca (T.A.); krussell@ualberta.ca (K.R.)

**Keywords:** ankle protection, ankle injury, basketball, injury prevention, Health Belief Model

## Abstract

This cross-sectional study explores factors affecting the decision of basketball players to wear ankle support devices (ASDs). A questionnaire regarding attitudes towards ASD usage was developed based on the Health Belief Model (HBM). The questionnaire assessed HBM perceptions (susceptibility, severity*,* benefits, and barriers) and modifying factors (demographic, personal history of ankle injury, influence of coach to preventive action) that may affect an athlete’s decision to wear ASDs. One hundred forty basketball players competing at the recreational, high school, or university levels completed the questionnaire, with the questionnaires being completed at the basketball gymnasium or at home. It was found that athletes whose coaches enforced ASD use were significantly more likely to wear them (OR: 35.71; 95% CI: 10.01, 127.36), as were athletes who perceived ankle injuries to be severe (OR: 2.77; 95% CI: 1.04, 7.37). Previous injury did not significantly increase the odds of using an ASD. The combined influence of coach enforcement and previous injury had the greatest effect on increasing ASD use. The largest barrier to ASD use was a lack of aesthetic appeal. Strategies aimed at increasing players’ willingness to wear ankle protection should be emphasized among coaches and parents as this may increase use of ASDs.

## 1. Introduction

Basketball is one of the most popular sports in the world with over 450 million documented participants playing organized basketball in 213 countries [1]. It is gaining global popularity and has replaced soccer as the most popular sport in Asia and Australia [1].

As the number of basketball players has increased so has the burden of injury, particularly amongst young men and women. From 2000 to 2001, basketball was the most frequent cause of sports-related emergency department visits for youth and adolescents in the United States with 395,251 reported cases [2]. Studies have identified ankle injuries as the most prevalent basketball-related injury [3,4,5] due to swift changes in direction while running, the frequent jumping and landing while shooting and rebounding, and the contact with other players [6,7]. Recovery from ankle injuries may result in missed time and residual symptoms, such as pain, instability, and weakness [8]. Other effects include medical expenses, decreased strength, delays in muscle reaction time, disability, and impaired athletic performance [3,8,9]. 

Ankle support devices (ASDs) include bracing or taping and are used to prevent initial ankle injury or repeat injury [3]. They have been shown to both reduce the incidence and severity of ankle injury, particularly for sprains [10,11,12,13,14]. A recent critical review of ankle sprain prevention suggests that ASDs along with neuromuscular training will achieve the best preventive outcomes [15]. Studies have demonstrated that ankle braces are more supportive, user friendly, and effective than tape [14,16,17]. Use of ankle braces results in a five-fold reduction in the incidence of ankle sprains among soccer players with previous injury [12]. Among college-aged intramural basketball players, taping reduces the incidence of ankle sprains to 14.7 sprains per 1000 player games compared with 32.8 sprains per 1000 player games found in the un-taped athletes [18]. However, common barriers to the use of protective equipment in relevant situations include feelings of discomfort and perceiving the ASD as unnecessary; in contrast, parental influence and younger age increase use [19,20]. It is unknown what specifically influences basketball players to use ASDs. A more behavioural approach towards injury prevention has previously been suggested [21]. 

The Health Belief Model (HBM) is a theoretical framework used to understand and predict health behaviors [22]. According to this model, the likelihood of an individual engaging in preventive health behavior can be predicted by four individual perceptions: perceived susceptibility to harm, perceived severity of injury, perceived benefits of the action, and perceived barriers to preventive action. A review of 46 studies utilizing the HBM identified perceived barriers to preventive action as the most consistent predictor of protective behavior [22]. The HBM has previously been used to study how psychological factors may affect protective gear use [23]. In this study, we aimed to understand the factors that affect a young basketball player’s decision to wear an ASD by way of an HBM guided questionnaire. 

## 2. Methods

### 2.1. Questionnaire Development

We initially interviewed 6 athletes, 2 coaches, 1 sport medicine physician, 1 physiotherapist, and 2 athletic trainers regarding ASDs and barriers to their use. To guide the open-ended discussion process, we asked: “What do you think influences an athlete’s decision to wear ankle protection?” If required, the question was rephrased: “Why do some basketball players choose to not wear ankle protection?” The responses were recorded and analyzed for recurring themes, which were then used to construct the questionnaire. 

The psychosocial variables identified as potentially influencing basketball players’ decisions to wear ankle protection included: cost of the ASD, impact of appearance with respect to social identity and image, degree of perceived physical comfort/discomfort, perceived negative effects on athletic performance, and coaches’ enforcement (*i.e.*, coaches may force their players to wear an ASD). 

As per the HBM, perceptions and modifying factors were also examined. Questions to represent each perception in the HBM were developed along with questions to identify modifying factors. Perceptions and their corresponding questions are presented in Table 1. The modifying factors that were identified with questions were demographics, personal history of self-reported ankle injury, and the influence of the coach.

**Table 1:** Health Belief Model (HBM) perceptions examined in questionnaire using corresponding questions.

Perception	Corresponding question
Perceived Susceptibility	Do athletes believe that playing basketball at their current level of competition exposes them to a greater risk of ankle injury?
Perceived Severity	Do athletes believe that ankle injuries require a significant amount of recovery time?
Perceived Benefits	Do athletes believe that ankle injury can be prevented by wearing ASDs?
Perceived Barriers	Do athletes believe that there are barriers preventing them from using ASDs?

The initial questionnaire was pilot tested with 6 players from recreational, high school, and university levels. A 5-point Likert-scale was used to quantify each of the four perceptions that influence ASD usage according to the HBM. The final questionnaire used in the study examined the level of perceptions of susceptibility (2 questions), severity (2 questions), and benefit (3 questions), demographic factors (2 questions), and personal history of ankle injury and ASD use (4 questions). Perceived barriers to use were also assessed: appearance (3 questions), performance (1 question), influence of professional athletes (2 questions), comfort (3 questions), and cost (3 questions).

### 2.2. Data Collection

The self-report questionnaire was administered by a research assistant to 94 male and 46 female basketball players in one recreational league (ages 11–20), two high school teams (ages 13–21), and three university-varsity teams (ages 18–26). All participants resided in the Toronto area. A research assistant attended the high school and university practices and administered the survey on site. Recreational players were given the survey to complete at home and returned by mail to a research assistant. The questionnaire required approximately 5–10 min to complete. 

### 2.3. Statistical Analysis

A Likert score of 4 (somewhat agree) and 5 (strongly agree) were defined as important and a Likert score of 1, 2, or 3 was deemed not important. For perceptions that had more than one question (e.g., severity of ankle injuries), a player had to respond with a 4 or 5 for all relevant questions in order for the perception to be classified as important. A chi-square omnibus test was used to determine if there was a significant difference in the proportion of athletes identifying a perception as important between the three levels of players. If a significant difference was observed (*p* < 0.05), additional chi-square tests were performed to determine where the difference occurred. To examine the various barriers, the analysis was repeated among the players who indicated that they did not currently use ASDs.

Forwards selection logistic regression was used to determine which variables (perceptions, coach enforcement, previous ankle injury) best predicted current ASD use. The variable that resulted in the smallest *p*-value was included in the model first and each additional variable was added one at time. The variable that was the most statistically significant (indicated by the *p*-value) was retained in the model and this process was repeated until there were 10 ASD users per included variable [24]. STATA 12 software was used to conduct all analyses. 

### 2.4. Ethics

Ethical approval was granted by the St Michael’s Hospital Review Board.

## 3. Results

In total, 140 athletes completed the survey, resulting in a response rate of 76.1%. Demographic data for the participants is summarized in Table 2. Overall, 32.9% of players wore an ASD and 81.4% self-reported a previous ankle injury. Compared with recreational and high school players, a significantly greater proportion of university players wore ASDs (*p* < 0.01) and had coaches who enforced ASD use (*p* < 0.01). There was no significant difference in previous ankle injury among the three levels of play. 

**Table 2:** Demographic characteristics of recreational, high school, and university basketball players.

	Recreational	High school	University	Overall
	*n* = 56 (%)	*n* = 32 (%)	*n* = 52 (%)	*n* = 140 (%)
Males (%)	56 (100)	19 (59.3)	19 (36.53)	94 (67.1)
Age (years) (mean ± SD)	14.9 ± 2.0	16.1 ± 1.7	20.6 ± 2.0	17.3 ± 3.3
Use ASD (%)	8 (14.3)	7 (21.9)	31 (59.6) *	46 (32.9)
Previous ankle injury (%)	41 (73.2)	28 (87.5)	45 (86.5)	114 (81.4)
Coach enforcement of ASD (%)	4 (7.1)	4 (12.5)	24 (46.2) *	32 (22.9)

ASD: ankle support device; * Significantly greater among university players versus recreational or high school players (*p* < 0.01).

The perceptions relevant to the HBM are presented in Table 3, and are stratified by competition level. Overall, the perceived susceptibility to ankle injury was the most frequently reported perception, with 34.3% of all athletes perceiving basketball as an activity with a heightened risk of ankle injury. Ankle injuries were perceived to be severe injuries by 44.3% of athletes, and 48.1% perceived ASDs as beneficial. In comparing the different competition levels, university athletes were significantly most likely to perceive ankle injuries to be severe (*p* < 0.01) and ASDs to be beneficial (*p* < 0.01). There was no significant difference in perceptions between recreational players and high school players. High school athletes were the least likely to perceive that they would benefit from ASDs at 18.8%. 

**Table 3:** Perceptions regarding ankle support devices (ASD) usage among recreational, high school, and university basketball players.

	Recreational	High school	University	Overall
	*n* = 56 (%)	*n* = 32 (%)	*n* = 52 (%)	*n* = 140 (%)
Perceive susceptibility to ankle injury	18 (32.1)	7 (21.9)	13 (25.0)	48 (34.3)
Perceive severity of ankle injury	18 (32.1)	12 (37.5)	32 (61.5) *	62 (44.3)
Perceive benefits of ASDs	14 (25.0)	6 (18.8)	25 (48.1) *	45 (32.1)

ASD: ankle support device; Based on defining “strongly agree” or “somewhat agree” as “important” to the individual; * Significantly greater among university players versus recreational or high school players (*p* < 0.01).

Athletes’ perceived barriers to ASD use are presented in Table 4, stratified by competition level. Overall, 53% of athletes reported at least one barrier to ASD use and the proportion of players reporting a barrier increased by level of competition—high school and university athletes reported a significantly higher proportion of at least one barrier to ASD use as compared to recreational players (*p* < 0.01). Among all level of players, aesthetic appearances was the most frequently reported barrier to ASD use (29.3%). Cost (12.1%) and comfort (15.0%) were the most infrequently reported barriers. When limited to the 94 athletes reported that they did not use ASDs, the perceived barriers in decreasing order were: aesthetic appearances (27.7%), appearance of weakness (20.2%), performance (17.0%), comfort (11.7%), and cost (6.4%).

**Table 4:** Reported barriers to ASD usage among recreational, high school, and university basketball players.

	Recreational	High school	University	Overall
	*n* = 56 (%)	*n* = 32 (%)	*n* = 52 (%)	*n* = 140 (%)
Any barrier	17(30.4)	19 (59.4) *	38 (73.1) *	74 (52.9)
Comfort	7 (12.5)	3 (9.4)	11 (21.2)	21 (15.0)
Performance	9 (16.1)	8 (25.0)	9 (17.3)	26(18.6)
Aesthetic appearance	12 (21.4)	11 (34.3)	18 (34.6)	41 (29.3)
Cost	0 **	6 (18.9)	11 (21.2)	17 (12.1)
Appearance of weakness	6 (10.7)	10 (31.3)	11 (21.2)	27 (19.3)

ASD: ankle support device; Based on defining “strongly agree” or “somewhat agree” as “important” barrier to player; * Significantly higher among high school and university players than recreational players (*p* < 0.01); ** Significantly lower among recreational players versus high school or university players (*p* < 0.01).

Athletes whose coaches enforced ankle protection were more than five times as likely to use ASDs (87.5% *vs.* 16.7%, *p* < 0.001). All university players who reported that their coaches enforced ASD usage also reported wearing ASDs. In addition, basketball players with a previous ankle injury were more than three times likely to use ASDs than those with no history of ankle injury (37.7% *vs.* 11.5%, *p* = 0.01). An additive effect between coach enforcement and previous injury was also observed. Athletes who had a previous ankle injury and were made to use ASDs by their coaches (89.7%) were significantly more likely (*p* < 0.001) to wear ASDs compared with previously injured athletes with no coach enforcement (20.0%) and athletes with neither coach enforcement nor a history of ankle injury (4.4%). In total, 48.6% of athletes believed that NBA and national team players wore ASDs and this did not differ by level of competition (*p* = 0.116). 

Several factors predicted ASD usage (Table 5). Athletes had significantly higher odds of wearing ASDs when their coach enforced usage (OR: 35.71; 95% CI: 10.01, 127.36). Athletes who identified cost as a barrier to ASD usage had significantly higher odds of ASD use (OR: 4.66; 95% CI: 1.13, 19.05). There was a significantly increased odds of ASD use among players who perceived ankle injuries to be severe compared with those who did not (OR: 2.77; 95% CI: 1.04. 7.37). The odds of ASD use were higher among players who had sustained a previous injury, although it was not statistically significant (OR: 3.93; 95% CI: 0.81, 19.03).

**Table 5:** The association between factors that predict ASD usage and actual ASD usage (odds ratios and 95% CI).

ASD usage	Odds ratio (95% CI)
Coach enforcement	35.71 (10.01, 127.36)
Cost barrier	4.66 (1.13, 19.05)
Perceive severity of ankle injury	2.77 (1.04, 7.37)
Previous ankle injury	3.93 (0.81, 19.03)

ASD: ankle support device.

## 4. Discussion

The odds of wearing an ASD while playing basketball was significantly higher among those who believed ankle injuries were of a severe nature and ASDs were effective. Additionally, coach enforcement was a significant predictor. The most common reason for not wearing an ASD was because they were aesthetically unappealing. 

Meeuwisse *et al*. [25] found that on average all ankle injuries resulted in a loss of 5.5 basketball sessions (games or practices) per injury [25]. Tears to the lateral collateral ligament may require the individual to wear a hinged knee brace or to even undergo surgical treatment [26]. However, 41% of respondents in our study did not consider ankle injuries to be severe. Athletes who perceived ankle injuries as severe were at a greater odds of wearing ankle protection than those who did not. Therefore, player, coach and parent education regarding the severity of ankle injuries may be important in increasing ASD use.

Previously established programs such as the Heads Up Hockey Program (an American ice hockey safety initiative) and Smart Hockey (a Canadian ice hockey injury prevention initiative) emphasize the importance of the coach as a role model who demonstrates respect for teammates, opponents, and the game [27,28]. Hockey coaches at the Atom level reported that most effective way to change player behavior was to change their coaching behavior [27,28]. The coach can be the single most important figure in terms of safety promotion—especially when they can mold a young player’s outlook on sport [29]. A role for coaches in endorsing ASD usage has been recommended [14]. Our study showed that players who had coaches who enforced ASD had greater usage, indicating that coaches may serve as a primary force in affecting ASD use and modifying sporting culture. Few recreational and high school players reported that their coaches enforced ASD usage and this is an opportunity for injury prevention programs to inform coaches about the effectiveness of ASDs. New Zealand Netball and Football (soccer) community-level coaches received an injury prevention curriculum as part of their education and 89% of netball and 96% of football coaches reported that the curriculum changed their coaching practices [30]. A coach taught injury prevention program resulted in a significant reduction in youth basketball acute injuries [31]. Therefore, coaches can play a valuable role in injury prevention. 

Having sustained an ankle injury is a risk factor for subsequent injury [18]. It would be expected that players who had a previous ankle injury would perceive these injuries to be severe compared with players with no previous ankle injury; however, there was no significant difference in perceptions of severity by history of previous injury. The majority of athletes with a prior history of ankle injury felt that ASDs prevented subsequent damage. It has been found that ASDs may be more effective in preventing re-injury than initial injury [12,15]. 

A significantly higher proportion of university players believed ASDs were beneficial. The HBM predicts that players who believe that ankle protection is effective would be more likely to use ASDs [22]. ASDs can decrease the predisposition of ankle inversion by stiffening the ankle joint to reduce range of motion and preventing the athlete from landing on an inverted foot [17,32]. ASDs may also increase an athlete’s proprioception, thus allowing a greater degree of muscular control [33]. Furthermore, ASDs can also serve as a psychological reminder to the athlete to moderate lower-limb behavior [32]. Our results showed that those who perceived ASDs as beneficial were 1.8 times more likely to wear ankle protection.

The literature suggests that perception of the severity of a potential injury and the benefits of preventive action are insufficient to predict the actual use of preventive measures because a person at risk must also have a willingness to engage in pro-health behavior [22]. For example, Dutch pediatricians were aware of bicycle helmet effectiveness and the majority reported professional or personal experience with bicycle injuries, yet 94% did not wear a helmet [34]. We found that only 17% of recreational and high school athletes wore an ASD. 

## 5. Conclusions

This study highlights the critical role of the coach in encouraging ASD use. Organized basketball leagues and camps could provide incentives for ASD use and disincentives for coaches and teams not utilizing ASDs. Second, education is needed to understand the significance and severity of ankle injuries in players and protective effect of ASDs. High school players may underestimate the severity of ankle injuries and rarely use ASDs, and educational interventions should be directed towards both players and their coaches. Since changes in knowledge and attitudes generally precede changes in behaviour [22], these goals could be accomplished by mandatory educational or training sessions given to all players and coaches in organized basketball. Finally, opportunities exist for manufacturers and other stakeholders to address barriers to use such as comfort and cost, as well as placing more emphasis on aesthetic considerations.

## 6. Limitations and Future Directions

There were some limitations to this study. Although we had representation from 140 players from a limited number and variety of teams, a larger sample size would allow for a more in-depth exploration of beliefs, behaviours, and associated factors around the use of ASDs. In addition, while the high school and university-level players included male and female players, the recreational players were part of a male-only division. However, a player’s sex was not found to be a significant predictor of ASD use, and therefore it may not be a significant limitation. We did not stratify respondents in to whether they owned ASDs or not. Researchers found that factors responsible for not wearing a bicycle helmets were different for helmet owners (loss of the helmet or helmet not needed) than non-helmet owners (comfort and appearance) [35]. Although we asked about cost as a barrier, we did not determine a player’s or patent’s ability to afford or access ASDs. Interestingly, players who identified cost as a barrier had a significantly higher odds of ASD use. We also did not ask if mandatory ASD use would influence their willingness to play organized basketball.

Future studies should include a larger sample size that includes a variety of geographical locations, and cultural and economic conditions. As well, the influential roles of parents, coaches, trainers and other role models such as professional athletes should be examined, as they could play important roles in shifting attitudes towards a willingness to use ASDs. Factors that would influence non-ASD users to use ASDs should be determined. Finally, more economic evaluation of the value of ankle protectors is required. 
